# The development and validation of a prototype mobility tracker for assessing the life space mobility and activity participation of older adults

**DOI:** 10.1186/s12877-020-01649-x

**Published:** 2020-07-22

**Authors:** Soon Hoe Ho, Dion Piu Sern Tan, Pey June Tan, Ka Wei Ng, Zoe Zon Be Lim, Isabel Hui Leng Ng, Lok Hang Wong, Mimaika Luluina Ginting, Belinda Yuen, Ullal Jagadish Mallya, Mei Sian Chong, Chek Hooi Wong

**Affiliations:** 1 Geriatric Education and Research Institute Ltd, 2 Yishun Central 2, Singapore, 768024 Singapore; 2 NDR Medical Technology Pte Ltd, 75 Ayer Rajah Crescent #02-19, Singapore, 139953 Singapore; 3 Lee Kuan Yew Centre for Innovative Cities, Singapore University of Technology and Design, 8 Somapah Road, Singapore, 487372 Singapore; 4 Department of Geriatric Medicine, Khoo Teck Puat Hospital, 90 Yishun Central, Singapore, 768828 Singapore; 5 The Geriatric Practice, 38 Irrawaddy Road #09-21, Mount Elizabeth Novena Specialist Centre, Singapore, 329563 Singapore; 6 Health Services and Systems Research, Duke-NUS Medical School, 8 College Road, Singapore, 169857 Singapore

**Keywords:** Mobility tracker, Global positioning system, Radio-frequency identification, Accelerometer, Life space

## Abstract

**Background:**

There is increasing interest in examining the life space mobility and activity participation of older adults in the community using sensor technology. Objective data from these technologies may overcome the limitations of self-reported surveys especially in older adults with age-associated cognitive impairment. This paper describes the development and validation of a prototype hybrid mobility tracker for assessing life space mobility and out-of-home activities amongst 33 community-ambulant older adults in Singapore.

**Methods:**

A hybrid mobility tracker was developed by combining a passive Global Positioning System logger, tri-axial accelerometer and radio-frequency identification. Objective measures of life space, derived from 1 week of tracking data using Geographic Information Systems, were the maximum Euclidean distance from home (Max Euclid) and the area of the minimum convex polygon surrounding all GPS waypoints (MCP area). Out-of-home activities were quantified by visually identifying the total number of activity nodes, or places where participants spent ≥5 min, from mobility tracks. Self-reported measure of life space in 4 weeks was obtained using the University of Alabama at Birmingham Study of Life Space Assessment (UAB-LSA) questionnaire. Self-reported out-of-home activities were recorded daily in a travel diary for 1 week. Bivariate correlations were used to examine convergent validity between objective and subjective measures of life space and out-of-home activities.

**Results:**

The mean age of participants was 69.2 ± 7.1 years. The mean UAB-LSA total score was 79.1 ± 17.4. The median (range) Max Euclid was 2.44 km (0.26–7.50) per day, and the median (range) MCP area was 3.31 km^2^ (0.03–34.23) per day. The UAB-LSA total score had good correlation with Max Euclid (r = 0.51, *p* = 0.002), and moderate correlation with MCP area (r = 0.46, *p* = 0.007). The median (range) total number of activity nodes measured by tracker of 20 (8–47) per week had a good correlation with the total activity count recorded in the travel diaries of 15 (6–40) per week (r = 0.52, *p* = 0.002).

**Conclusions:**

The tracking system developed to understand out-of-home travel was feasible and reliable. Comparisons with the UAB-LSA and travel diaries showed that it provided reliable and valid spatiotemporal data to assess the life space mobility and activity participation of older adults.

## Background

The concept of life space refers to the geographic extent within which an individual lives and moves. It is an interaction between the individual and the physical environment, determined by the individual’s mobility and other intrinsic capacities, as well as environmental factors in either enabling or restricting such interactions [1–3]. In the context of ageing, a progressive decline of physical, functional and cognitive health limits an individual’s ability to overcome environmental constraints for mobility, thus constricting the life spaces of older adults and often leaving them more confined to their local neighbourhoods compared to people in other age groups [1, 3–5]. Studying the changes in older people’s life space mobility, therefore, renders valuable information for the understanding of their person-environment interactions within the real world and the community.

To date, the measurement of life space mobility is largely dependent on self-reported time-space travel and activity diaries. However, the accuracy of self-reported measures is limited by the user’s capacity to record their activities reliably [6, 7]. This presents a challenge particularly to older users who are at higher risk of cognitive impairment [8, 9]. In recent years, a variety of sensors have been developed to gather objective data on human mobility, including Radio-Frequency Identification technology (RFID), the Global Positioning System (GPS) and accelerometers, each having its own advantages and limitations [10].

The use of RFID and GPS is gaining popularity in health research seeking to uncover the relationships between geographical location and various health outcomes. Whilst RFID offers portability, with its tags easily tucked away in pockets or bags [9], its accuracy is dependent on the number of readers placed within a limited area for tracking. This makes studying a larger area difficult and costlier, as a large number of readers is required [9, 11]. GPS, on the other hand, is able to track locations over large areas on the earth using satellites [9]. The accuracy of spatial coordinates, however, depends on the reception of satellite signals of sufficient quality as signals could be obstructed by solid walls and structures such as in the underground, or by atmospheric conditions and electromagnetic waves. Nevertheless, GPS offers researchers the opportunity for continuous and intensive high-resolution data collection in time and space over long durations [12, 13]. In contrast to RFID and GPS, accelerometers do not capture spatial data. They detect acceleration and have been widely used in health studies to measure walking speeds, postural changes and other body movements [10].

In this study, we developed a hybrid system by combining these three technologies (GPS, accelerometer and RFID) for tracking the outdoor mobility and activity participation of older adults with no self-reported history of dementia within their life spaces for 1 week. Three technologies were used to counteract the limitations of a single technology, and a design-thinking approach was used to create a prototype mobility tracker that suits the mobility and usage patterns of older adults. The tracker was developed as a prototype in a national level project on planning and design of age-friendly neighbourhoods in Singapore. One of the aims of that larger project was to understand the daily habitual outdoor movements and activity patterns of older adults.

The paper describes the development of the prototype hybrid mobility tracker and systematically compares objective data from the tracker to self-reported measures to assess its validity and reliability in measuring life-space mobility and out-of-home activities in community-dwelling older adults. Convergent validity was established from bivariate correlations between objective measures of life-space mobility and out-of-home activities obtained from the mobility tracker, and subjective data obtained from the self-reported University of Alabama at Birmingham Study of Aging Life Space Assessment (UAB-LSA) [1, 2] and travel diaries [14, 15]. We also present a case study of using the tracker for 1 week in an older adult without dementia to demonstrate the tracker’s feasibility and validity.

## Methods

### Target research population

This was a prospective study based on cross-sectional design. The study recruited adults aged 55 years and above who were able to mobilise independently for a distance of approximately two bus stops (400 m), able to leave home at least once a week and had no self-reported history of dementia. A total of 33 community-dwelling older adults were recruited through a convenience sampling approach in community centres, senior care centres and senior activity centres in three neighbourhoods located in the western, central and eastern regions of Singapore from January to June 2018. Potential participants were invited to a face-to-face interview with members of the research team to understand the study components and provide informed consent before participating. The study was approved by the National Healthcare Group’s Domain Specific Review Board.

### Data collection

#### Baseline assessment

Baseline sociodemographic characteristics, including cognitive status based on the Mini-Mental State Examination (MMSE) [16], were collected through face-to-face interviews by a trained researcher in participants’ homes using their preferred language (English, Malay, or Mandarin). The Malay and Mandarin versions of the MMSE questionnaire were developed by forward- and backward-translations of the English questionnaire by independent translators.

#### Subjective life space and activity participation (UAB-LSA and travel diaries)

*Life space mobility* was measured at baseline using the University of Alabama at Birmingham Study of Aging Life Space Assessment questionnaire (UAB-LSA) [1, 2]. The UAB-LSA provides a total score accounting for (a) life space level reached in the past 4 weeks, where a higher level denotes a farther distance from home; (b) the frequency of travel; and (c) the level of mobility independence during the travel. A higher total score corresponds to a larger life space [1, 2]. The survey was administered by a trained researcher in participants’ homes using their preferred language (English, Malay, or Mandarin). The Malay and Mandarin versions were developed by forward- and backward-translations of the English questionnaire by independent translators.

*Activity participation* was tracked daily for 1 week, by using travel diaries with daily entries of the weather, places visited, time of visit, mode of transport and types of activities participated.

#### Objective life space and activity participation (prototype mobility tracker)

Participants were given a pocket-sized mobility tracker to be carried out-of-home every day for 1 week. *Life space mobility* was derived from the tracking data using two measures: (a) area of the minimum convex polygon (abbreviated MCP area) around all GPS waypoints, and (b) maximum Euclidean distance from home (abbreviated Max Euclid) [17].

*Activity participation* was estimated by identifying “activity nodes”, which were the significant places where participants had stopped for 5 minutes or more [9, 18]. Activity nodes were visually identified from the mobility tracks during the processing of raw GPS data.

### Development of the prototype mobility tracker

**Fig. 1:**
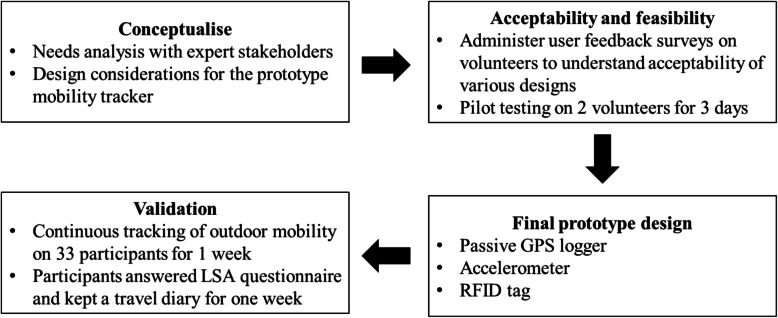
Study flow diagram

Fig. 1 illustrates the study flow from conceptualisation and development of the prototype mobility tracker to its validation.

#### Design considerations

The prototype was developed in consultation with geriatricians, engineers and researchers in the fields of aged-care and mobility. Prototype design considerations and hardware requirements are summarised in Table 1.

**Table 1:** Design considerations of the prototype mobility tracker

**Hardware requirements**	
• The system should incorporate:	
Passive radio-frequency identification (RFID) device or equivalent	
o To accurately detect when the user leaves and arrives home.	
o RFID reader should have algorithms to prevent false positives.	
Global Positioning System (GPS) logger or equivalent	
o To collect geospatial location and outdoor travel data every second.	
o Accuracy of at least 10 m.	
o Should be able to lock GPS position from cold start in less than 1 min.	
o Should be able to record distance and travel speed to decode travel mode.	
Accelerometer or equivalent	
o To validate GPS data and track movement out of home where GPS signal is unavailable.	
o The device should have a sampling rate that would enable it to detect falls as an adverse event (typically 5–8 Hz).	
**Data output requirements:**	
• The system should accurately track the following indicators:	
o Type of travel mode (i.e. walking, vehicular).	
o Time spent per travel mode (per day).	
o Total distance travelled from home (per day).	
o Total distance travelled per travel mode (per day).	
o No. of walking tracks (per day) (walking track identified by speed ≤5 km/h) [18, 19].	
o No. of steps (per day).	
o Time spent out of home (per day).	
o Time spent in location (per location per day).	
o Location (latitude and longitude in SVY21 projection).	
o No. of activity nodes (per day) (defined as places participants stayed for ≥5 min [9, 18].	
• Spatial format in latitude, longitude and fixed projection.	
• Retrieved location data should be exportable for geospatial mapping.	
• Retrieved walking data should be exportable in commonly used data formats.	
**Older adult-customised design/feasibility requirements**	
• The tracker should operate without additional input from users.	
• The tracker should be durable and resistant to adverse weather conditions.	
• The tracker should be small, lightweight, and non-intrusive or distractive.	
• The tracker should be safe to operate and socially acceptable to older adults.	
**Reliability requirements:**	
• The RFID reader should last continuously for at least 7 days.	
• The system should require minimal training to operate.	

#### Development and pilot testing for acceptability of GPS loggers and RFID system

User feedback surveys were conducted in volunteers aged ≥55 years during prototype development to understand the acceptability and usability of several wearable mobility tracker designs. Pilot testing of two different GPS loggers and the RFID system was conducted in two volunteers (aged 66 and 67 years old) for 3 days before finalising the prototype mobility tracker.

#### Final device

**Fig. 2:**
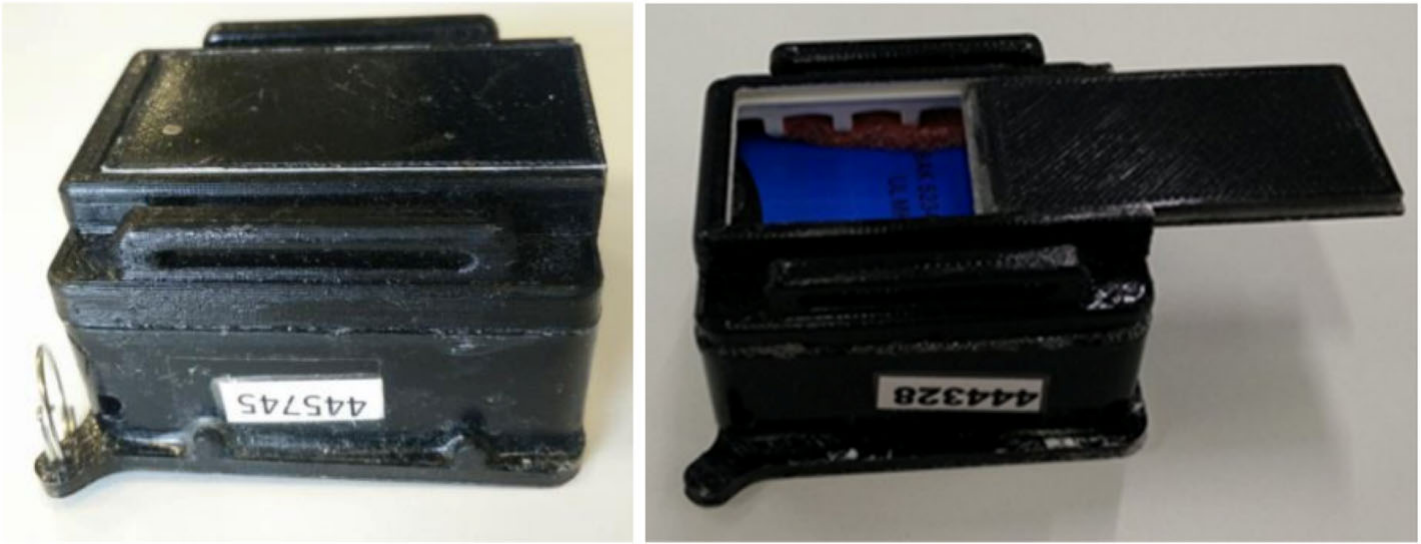
Casing containing GPS logger and accelerometer (left); back sliding cover for insertion of accelerometer (right)

The device consisted of an encased GPS logger and accelerometer, whilst the RFID tag was located externally for better sensitivity. It did not have a display screen in order to prevent participants from tracking their own activity, hence limiting cognitive bias. A hard casing was designed to protect the GPS logger and accelerometer from accidental tampering (Fig. 2).

The GPS logger was a passive logger which did not transmit any data (iTrail H6000 GPS logger) [20]. Instead, data was saved in its internal memory and downloaded for processing before being viewed on a mapping program (i.e. Google Maps). The accelerometer had a sampling rate of 12.5 Hz (i.e. capturing data 12.5 times every second) (Axivity AX3 3-axis logging accelerometer) [21]. It integrated the acceleration experienced in three coordinate axes to give an overall acceleration. A Python program was developed to calculate the number of steps and walking speed from the accelerometer’s raw data [22, 23]. More detailed description is available in the supplementary material [see Additional file 1].

The RFID system had a read range of three metres. The RFID reader (KL9001R) [24] was placed at the main door of participants’ homes at waist or shoulder level. Participants were instructed to place the RFID tag inside a pouch with a carabiner or a keychain. Each RFID tag was labelled with a unique identification number (ID) to allow for the tracking of multiple participants from the same home. A five-minute time difference was set for each RFID tag to prevent the detection of false positives whenever participants left their homes for less than 5 minutes (i.e. not considered an outdoor trip). The RFID system detected when the participant left and returned home. To improve adherence and ensure that participants remembered to bring the device whenever they travelled outside of home, reminder posters were placed at prominent positions near home entrances.

As a re-chargeable device was deemed unsuitable for older adults (who may forget to charge the device), the device was designed for a maximum battery life span of 4–5 days. In addition, the GPS logger was able to conserve its battery life by powering off when no motion was detected for 5 minutes. As such, the research team had to visit the homes of the participants only once (i.e. midway during the week-long data collection period) to exchange their existing device with another fully-charged device. This was to ensure that participants would always carry devices with sufficient battery power at all times. Raw data from the tracking system were processed and presented per participant per day, which were used for further analysis.

### Data analyses

Spatial data was derived using a Geographic Information System (GIS), QGIS version 2.18.13 [25]. Quantitative statistical analyses were performed on R version 3.3.1 [26]. Parametric data were reported as mean and standard deviation, whilst non-parametric data were reported as median and range. Convergent validity was assessed between objective and subjective measures of life space and activity participation, using bivariate correlations between the UAB-LSA Total score (LSA Total) and maximum Euclidean distance from home (Max Euclid), between LSA Total and the area of the minimum convex polygon surrounding all GPS waypoints (MCP area), and between the number of activity nodes detected from the mobility tracker and the number of activities recorded in the travel diary [14, 15]. Non-parametric Spearman rank correlation was used as variables were not normally distributed using Shapiro-Wilk test [15, 27]. Spearman correlation coefficients of 0.25–0.50 were moderate, 0.50–0.75 good, and > 0.75 excellent [15, 28]. The minimum sample size to detect a correlation coefficient of 0.50 with 80% power at two-tailed 5% is 29 [15, 29]. Statistical significance was taken as *p* ≤ 0.05.

## Results

### Participants’ sociodemographic characteristics

Thirty-three older adults completed the study. The mean age of the participants was 69.2 years (SD 7.1 years; range = 56–84 years), and 21 were women (63.6%). Half of the participants (45.5%) had primary-level education (i.e., 6 years or less of formal education). Seventeen (51.5%) individuals were married and two-thirds (69.7%) were retired with a mean retirement duration of 12.4 years. Eight individuals (24.2%) lived alone. The mean MMSE score was 26.9 (SD 2.2; range = 21–30) (Table 2).

**Table 2:** Sociodemographic characteristics of the study participants

Demographic variable (*N* = 33)	Mean (SD)/ count (%)
Age, mean (SD)	69.2 (7.1)
Female, n (%)	21 (63.6)
Ethnicity, n (%)	
Chinese	31 (93.9)
Malay	2 (6.1)
Highest education completed, n (%)	
No formal education	3 (9.1)
Primary school	15 (45.5)
Secondary school	8 (24.2)
Post-secondary (Polytechnic, ITE, Junior College)	3 (9.1)
Tertiary (University and post-graduate degree)	3 (9.1)
Don’t know / Not sure	1 (3.0)
Marital status, n (%)	
Single	6 (18.2)
Married	17 (51.5)
Widowed	6 (18.2)
Divorced	3 (9.1)
Others	1 (3.0)
Employment status, n (%)	
Employed part time	6 (18.2)
Unemployed	2 (6.1)
Retired	23 (69.7)
Others	2 (6.1)
Number of years retired, mean (SD)	12.4 (8.7)
Housing type, n (%)	
Public housing (HDB) 1–2 room	3 (9.1)
Public housing (HDB) 3 room	15 (45.5)
Public housing (HDB) 4 room	9 (27.3)
Public housing (HDB) 5 room / Housing and Urban Development Company (HUDC) flats / Executive flat	5 (15.2)
Private housing (Condominium / Apartment)	1 (3.0)
Number of years lived in the neighbourhood, mean (SD)	23.1 (15.2)
Living alone, n (%)	8 (24.2)
Mini-Mental State Examination Score, mean (SD)	26.9 (2.2)

### Life space mobility and activity participation

The total number of participant-days recorded in the 33 travel diaries was 215 days, which was 98.6% of the total number of participant-days tracked by the mobility tracker (218 days) (Table 3). Activities recorded in the travel diaries include active leisure, attending educational courses, grocery shopping, and dining out. No participant reported any difficulty in using the mobility tracking device.

**Table 3:** Life space mobility and activity participation of study participants in one week^a^

**Measure of life space extent (N = 33)**	**Data source**	**Mean (SD)/ Median (range)**
Max Euclid per day (km), median (range)	Mobility tracker	2.44 (0.26–7.50)
MCP area per day (km^2^), median (range)	Mobility tracker	3.31 (0.03–34.23)
UAB-LSA total score (maximum score 120), mean (SD)	Questionnaire	79.1 (17.4)
**Activity participation (N = 33)**	**Data source**	**Mean (SD)/ Median (range)**
Total number of activity nodes per week, median (range)	Mobility tracker	20 (8–47)
Total number of activities per week, median (range)	Travel diary	15 (6–40)
Average time spent per activity node (minutes), median (range)	Mobility tracker	46.59 (26.20–259.00)

^a^ Data is reported as mean (SD) if normally distributed, and median (range) if not normally distributed, based on the Shapiro-Wilk test. Legend: *MCP Area* Area of the minimum convex polygon around all GPS waypoints, *Max Euclid* Maximum Euclidean distance from home, *UAB-LSA* University of Alabama at Birmingham Study of Aging Life Space Assessment

**Fig. 3:**
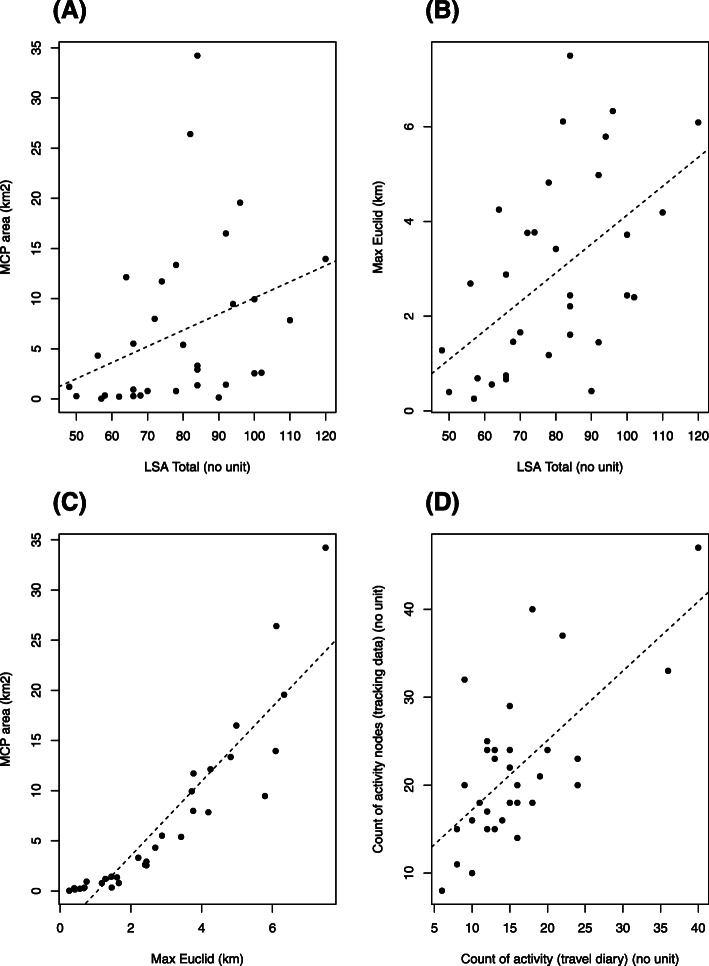
Relationships between objective and subjective measures of life space mobility and activity participation

Bivariate plots between objective and subjective measures of life space and outdoor activities are shown in Fig. 3. The mean UAB-LSA total score was 79.1 ± 17.4. Participants travelled from home a median (range) Max Euclid of 2.44 km (0.26–7.50) per day. The median (range) MCP area was 3.31 km^2^ (0.03–34.23) per day. Participants’ UAB-LSA total scores had a good correlation with their average daily Max Euclid (r = 0.51, *p* = 0.002). The correlation of their UAB-LSA total scores with their average daily MCP area was moderate (r = 0.46, *p* = 0.007).

For out-of-home activities, the median (range) total number of activity nodes measured by the tracker was 20 (8–47) per week, and the median (range) total activity count recorded by travel diaries was 15 (6–40) per week. The total number of activity nodes per week detected by the mobility tracker had a good correlation with the total number of activities recorded in the travel diary (r = 0.52, *p* = 0.002).

### Demonstration

#### Case study: detailed travel mobility and activity of one participant for 1 week

The detailed travel mobility and activity of one participant to demonstrate the use of the prototype mobility tracker in a real world setting is presented in Table 4. The mode of travel was inferred from the speed recorded by the GPS logger, in which speeds ≤5 km/h were walking speeds [18, 19]. It was shown that places frequented within the neighbourhood included food and beverage outlets, commercial establishments, wet markets, exercise facilities, community centres and parks. These were congruent with data from the travel diary. On average, the participant spent 9.6 h out-of-home, took 11,914 steps, walked 3.9 km, had an average outdoor gait speed of 3.72 km/h (1.03 m/s), and travelled 10.3 km by vehicle per day.

**Table 4:** Travel record of a participant for one week

Day	Place	Travel Mode	RFID Reach Home Time	RFID Leave Home Time	Walking distance (km)	Speed (km/h)	Total Vehicle Travel Distance (km)	Time Spent out of Home (s)	Total Number of Steps
1	Home	–	–	11:40	2.7	3.3	–	19,626	7854
	CC^a^	Walk							
	RC^b^	Walk							
	Home	Walk	17:07						
2	Home	–	–	6:13	6.4	3.53	–	42,661	16,648
	Housing Block	Walk							
	Home		7:40	7:55					
	Housing Block	Walk							
	RC^b^	Walk							
	CC^a^	Walk							
	Stadium	Walk							
	Town Centre	Walk							
	Home	Walk	18:20	–					
3	Home	–	–	6:15	3.8	3.02	51.3	56,019	14,197
	Stadium	Walk							
	Housing Block	Walk							
	RC^b^	Walk							
	MRT^c^	Vehicle							
	Shopping centre	Vehicle							
	Housing Block	Vehicle							
	Supermarket	Vehicle							
	Town Centre	Vehicle							
	Home	Vehicle	21:49	–					
4	Home	–	–	7:15	6.6	3.96	–	32,393	17,842
	RC^b^	Walk							
	Market	Walk	7:55	9:27					
	Association	Walk							
	Home	Walk	11:44	13:04					
	Housing Block	Walk							
	Housing Block	Walk							
	Stadium	Walk							
	Sports Complex	Walk							
	Home	Walk	17:35	–					
5	Home	–	–	6:22	1.9	4.12	8.9	30,150	7559
	Housing Block	Walk							
	Housing Block	Walk							
	Home	Walk	8:27	11:32					
	RC^b^	Walk							
	Relative’s House	Vehicle							
6	Relative’s House	–			2.3	4.0	12	34,820	6616
	Green Park	Walk							
	Housing Block	Walk							
	Housing Block	Walk							
	RC^b^	Walk							
	Home	Walk	17:45	–					
7	Home	–	–	11:13	3.5	4.13	–	25,266	12,684
	RC^b^	Walk							
	Housing Block	Walk							
	Home	Walk	16:08	17:11					
	Frozen yogurt shop	Walk							
	Fast food restaurant	Walk							
	Café	Walk							
	Home	Walk	19:17	–					

Abbreviations: ^a^*CC* Community Club, ^b^*RC* Residents’ Committee, ^c^*MRT* Mass Rapid Transit

## Discussion

The mobility tracking system using a combination of three technologies was both feasible and reliable in tracking older adults’ out-of-home movements. There was high accuracy in the spatial coordinates and timestamps recorded by the GPS logger when the person was out-of-home. When the GPS data was triangulated with accelerometer data, detailed information of the real-world outdoor mobility and travel patterns was obtained.

This study demonstrates the potential for using a mobility tracker to measure the life space mobility of older adults. Understanding life space mobility is important as it is an indicator of an individual’s intrinsic capacity and is reflective of quality of life [5, 30]. When examining the convergent validity between the objective and subjective measures of life space, there was good correlation between UAB-LSA total score and Max Euclid and moderate correlation between UAB-LSA total score and MCP area. The UAB-LSA questionnaire was used for convergent validity as it is a reliable measure of self-reported life space mobility [1, 2], and to ensure comparability with other studies. The moderate to good correlations could be due to the duration of recall as UAB-LSA required participants to recall over a four-week period [1, 2] while the objective GPS data was generated over 1 week. Physical activity tracking studies usually perform continuous tracking for at least 1 week for representative data [31]. Using a tracker to monitor individuals for 1 month is costly, manpower-intensive, and comes with an increased risk of non-compliance [32]. There is currently a lack of comparable survey instruments to measure life space mobility over a one-week period for community-dwelling older adults [33].

An inherent limitation of the UAB-LSA is the potential for recall bias. A study amongst healthy older adults in Canada have observed statistically significant correlations of 0.30–0.40 between objective and self-reported measures of life space, obtained from a GPS-cum-accelerometer device and the UAB-LSA respectively [34]. The fact that our study found stronger correlations can possibly be attributed to several factors, such as the different objective measures of life space derived from the data, our shorter tracking period of 1 week, and higher compliance by our participants. Self-reported measures of life space have also been shown to be underestimated compared to sensor-driven indicators [35]. These suggest that a self-reported measure like the UAB-LSA questionnaire may not adequately capture community mobility and activity recorded by the tracking device.

A good correlation was found between the number of out-of-home activities recorded in the travel diaries and the number of activity nodes identified by the tracking device. This study provides quantitative evidence of the feasibility of a GPS-enabled tracking device to capture more instances of destinations for activities compared to that reported in the travel diaries. However, the data also shows evidence of a gap in the number of activities unreported in travel diaries but detected by the mobility tracker. It was suggested that some destinations are perceived as more relevant or significant by older adults due to characteristics like purpose of trip, variety and availability of resources, and may be more likely to be recorded in self-reported diaries, which should be explored in future research [36, 37]. Other discrepancies between travel diaries and objective sources like GPS tracking have been attributed to trip “chaining” where individuals lump several short trips into one, and the tendency to record round numbers [38]. Whilst both tools are equally useful for understanding the outdoor activities of individuals, each has its strengths and limitations. Travel diaries document the types of activities performed and other qualitative information which are unavailable from the mobility tracker. On the other hand, information from the tracker is more accurate spatially and objectively with no recall bias.

None of our participants experienced any problem with the mobility tracker. While mild cognitive impairment might affect the complexity of travel, it did not hinder any participant from using the mobility tracker outside the home. There are several advantages in using the mobility tracker to study the life space and activity participation of older adults. Firstly, it overcomes the limitation of recall bias as experienced in the use of a survey instrument. Secondly, the measure of life space is not subjected to individual perceptions of what constitutes the “neighbourhood” and “town”, which is required for survey instruments in defining different life space levels. The objective distance-based measure of life space directly removes the need to differentiate “neighbourhood” from “town”, which might have different meanings for different people, and for those living in urban or rural settings [39]. This problem would lead to inferential biases, hence life space measures based on the UAB-LSA may not be generalizable across studies from different rural or urban centres and countries [40]. Thirdly, the objective data analysed on GIS enabled detailed visualization and quantification of out-of-home movement and travel to increase the understanding of spatiotemporal behaviour of older adults in their environment, hence providing valuable insight to ageing research on person-environment interactions [4].

The development and design of a mobility tracking system demands a user-centric approach. In our study, end-user surveys were administered to understand the acceptability and usability of different wearable mobility tracker designs amongst older adults, in order to increase compliance with wearing a tracking device. One additional consideration during the design process was the battery life of the device. A long battery life was especially important to allow the use of such devices in older adults with cognitive impairment [8].

Modern digital sensing technologies open up new ways to understand human mobility, allowing for a linkage of that information with location and other geographic information. They offer real-time objective readings of individual mobility as compared to survey instruments, travel diaries, and interviews, and are usually more accurate and granular than the latter methods [2, 41]. When entered into a spatial analysis program like GIS, they extend our understanding of space and time usage by individuals at various locations in greater detail [42]. Using these technologies in cities like Singapore, however, is not without limitations. For instance, GPS loggers are unable to access radio waves from orbiting satellites inside buildings or the underground. In consequence, there could be gaps in spatial coordinates when users travel in underground trains, for example. Advanced technologies, though useful in mapping out-of-home mobility and locations, do not fully replace questionnaires, diaries or interviews, which remain important methods to gather in-depth information about older adults’ travel behaviour and outdoor activities.

## Conclusion

This is, to our knowledge, the first prototype hybrid mobility tracking system comprising of a GPS logger, an accelerometer, and RFID that was developed for measuring the life space, mobility and activity participation of community-dwelling older adults. This study establishes its validity and reliability, with moderate to good correlations between objective tracking data and the self-reported life space and activities reported in the UAB-LSA questionnaire and travel diaries. We found that objective data provided better quantification of the multidimensional aspects of life space extent and out-of-home activities compared to the subjective methods as it is less prone to recall bias. When supplemented with travel diaries and a life space questionnaire, one would gain a richer understanding of older adults’ travel and activity patterns outside the home. Therefore, future community outdoor mobility studies could employ both objective and subjective methods to gather in-depth information on individual travel patterns and behaviour. As the trend in global aging increases, a better understanding of older persons’ outdoor mobility could help in tailoring programmes and city planning toward the needs of older adults [3, 43, 44].

## Supplementary information

**Additional file 1.** Description of the algorithms to calculate steps and walking speed. A brief description of the algorithms used to calculate the number of steps and walking speed of the participants from the raw accelerometer data. Two diagrams are included for illustration.

## Data Availability

The raw datasets generated and/or analysed during the current study are not publicly available due to a confidentiality agreement between the research parties.

## Abbreviations

GPS: Global Positioning System
GIS: Geographic Information Systems
UAB-LSA: University of Alabama at Birmingham Study of Aging Life Space Assessment
RFID: Radio-frequency identification
Max Euclid: Maximum Euclidean distance from home
MCP area: Area of the minimum convex polygon
LSA Total: UAB-LSA total score
