# Supplementary material for: The development and validation of a prototype mobility tracker for assessing the life space mobility and activity participation of older adults
Source: BMC Geriatr. 2020 Jul 22;20:251. doi: 10.1186/s12877-020-01649-x (PMC7374961; doi:10.1186/s12877-020-01649-x)
Supplement: Supplementary file 1 — Additional file 1. Description of the algorithms to calculate steps and walking speed. A brief description of the algorithms used to calculate the number of steps and walking speed of the participants from the raw accelerometer data. Two diagrams are included for illustration. [file 12877_2020_1649_MOESM1_ESM.pdf]

Additional File 1

Description of the algorithms to calculate steps and walking speed

Step Counter

The net acceleration is calculated by combining the accelerations experienced in the three axes and is then plotted on a graph. The "peaks" in the magnitude of net acceleration are considered as "steps" taken by the user. Generally, a "peak" is counted by using three consecutive points (Start Point, Max Point, and End Point) which follow the pattern: Start Point < Max Point > End Point (Figure S1).

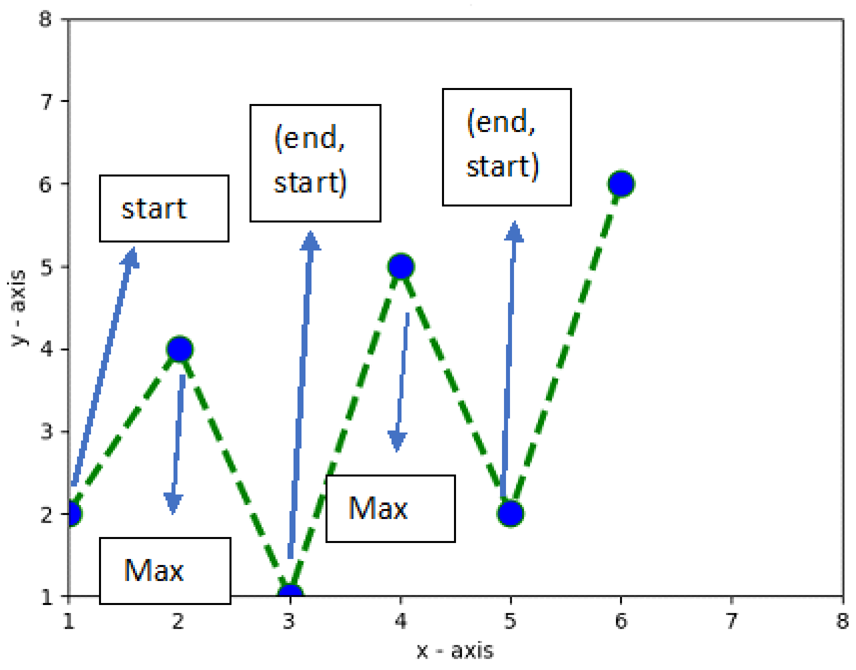

Fig S1: Illustration of method to identify "peaks" (Max Points) in the acceleration data.

Two threshold values (generally set by the standard deviation of the data, adjusted according to how the device was worn by the user) were set to filter out false positives. Peaks which lie above the maximum or below the minimum thresholds were not counted as steps (Figure S2).

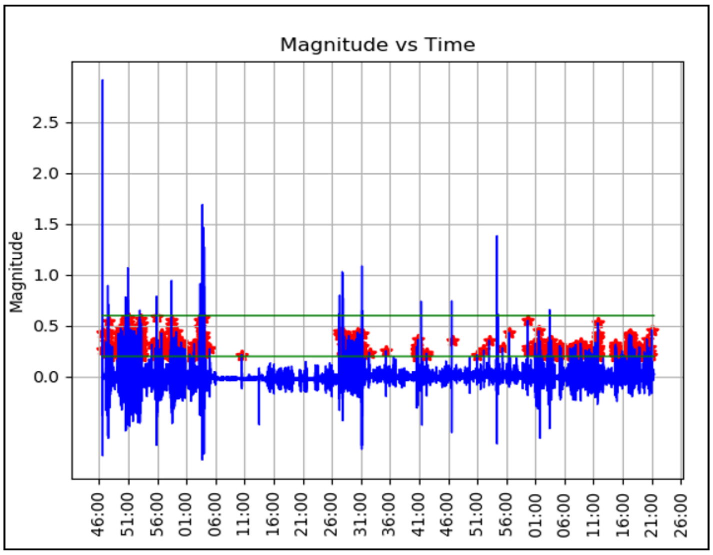

Figure S2: “Peaks” that fall between the threshold values were counted as steps.

Walking Speed

Walking speed is derived from acceleration via the formula: $v_f = at - v_i$, where a is the acceleration, v_f is the final speed after an interval of time t, and v_i is the initial speed. Walking speed is assumed to be 0 m/s when the step count is zero.
